# Targeting of Janus Kinases Limits Pro-Inflammatory but Also Immunosuppressive Circuits in the Crosstalk between Synovial Fibroblasts and Lymphocytes

**DOI:** 10.3390/biomedicines9101413

**Published:** 2021-10-08

**Authors:** Nina Yao, Theresa Tretter, Peter Kvacskay, Wolfgang Merkt, Norbert Blank, Hanns-Martin Lorenz, Lars-Oliver Tykocinski

**Affiliations:** Department of Medicine V, Division of Rheumatology, University of Heidelberg, D-69120 Heidelberg, Germany; nina.yao@med.uni-heidelberg.de (N.Y.); theresa.tretter@med.uni-heidelberg.de (T.T.); peter.kvacskay@med.uni-heidelberg.de (P.K.); wolfgang.merkt@med.uni-heidelberg.de (W.M.); norbert.blank@med.uni-heidelberg.de (N.B.); Hanns-Martin.Lorenz@med.uni-heidelberg.de (H.-M.L.)

**Keywords:** janus kinases, JAK inhibitors, tofacitinib, baricitinib, upadacitinib, rheumatoid arthritis, synovial fibroblasts, T helper cells, bDMARDs

## Abstract

Crosstalk between synovial fibroblasts (SF) and immune cells plays a central role in the development of rheumatoid arthritis (RA). Janus kinase inhibitors (JAKi) have proven efficacy in the treatment of RA, although clinical responses are heterogeneous. Currently, little is known regarding how JAKi affect pro- and anti-inflammatory circuits in the bidirectional interplay between SF and immune cells. Here, we examined the effects of tofacitinib, baricitinib and upadacitinib on crosstalk between SF and T or B lymphocytes in vitro and compared them with those of biologic disease modifying anti-rheumatic drugs (bDMARDs). JAKi dose-dependently suppressed cytokine secretion of T helper (Th) cells and decreased interleukin (IL)-6 and matrix metalloproteinase (MMP)3 secretion of SF stimulated by Th cells. Importantly, JAK inhibition attenuated the enhanced memory response of chronically stimulated SF. Vice versa, JAKi reduced the indoleamine-2,3-dioxygenase (IDO)1-mediated suppression of T cell-proliferation by SF. Remarkably, certain bDMARDs were as efficient as JAKi in suppressing the IL-6 and MMP3 secretion of SF stimulated by Th (adalimumab, secukinumab) or B cells (canakinumab) and combining bDMARDs with JAKi had synergistic effects. In conclusion, JAKi limit pro-inflammatory circuits in the crosstalk between SF and lymphocytes; however, they also weaken the immunosuppressive functions of SF. Both effects were dose-dependent and may contribute to heterogeneity in clinical response to treatment.

## 1. Introduction

Rheumatoid Arthritis (RA) is a systemic autoimmune disease characterized by chronic inflammation and articular joint destruction, affecting approximately 1% of the adult population worldwide. If insufficiently controlled, RA can lead to progressive disability, resulting in significant reduction in quality of life and high socio-economic costs. Multiple inflammation-associated secondary co-morbidities in RA lead to a shortened life expectancy [1,2].

Continuous medical developments have dramatically improved the outcomes for patients with RA. Traditional therapeutic approaches have relied on glucocorticoids, nonsteroidal anti-inflammatory drugs (NSAIDs) and small molecule disease modifying anti-rheumatic drugs (DMARDs) such as methotrexate, sulfasalazine or leflunomide. Glucocorticoids and NSAIDs interfere with the inflammatory cascades while DMARDs impede both the inflammatory and the destructive processes of RA [1,3]. These drugs, although efficient, are not specifically directed against inflammatory cells or cytokines and are associated with significant toxicity.

The development of biologic DMARDs (bDMARDs), such as monoclonal antibodies or recombinant soluble receptors, has been an important step forward. Their ability to neutralize specific cytokines or target distinct immune cells filled a gap in existing treatment options for RA and other autoimmune diseases. BDMARDs targeting tumor necrosis factor alpha (TNFα) were the first to be approved for the treatment of RA, followed by bDMARDs targeting interleukin (IL)-1beta or the IL-6 receptor. All have proven clinical efficacy, demonstrating the critical role of cytokines in the pathogenesis of RA [3,4]. However, some patients still have only partial or no response to bDMARDs, and sustained remission is rarely achieved.

More recently, a group of chemical entities has been developed that inhibit the janus kinase (JAK) family of intracellular tyrosine kinases, which transmit cytokine-mediated signals via the JAK-signal transducer and activator of transcription (STAT) pathway [5]. In mammals, four different JAK isotypes—JAK1-3, and tyrosine kinase 2 (TYK2)—have been identified, each associated with distinct cytokine receptors and different preferences regarding phosphorylation of specific subsets of STATs [6]. Tofacitinib was the first JAK inhibitor (JAKi) approved for the treatment of RA by the FDA in 2012 and by the EMA in 2017. Tofacitinib exhibits a selectivity to inhibit JAK1 and 3 and to a lesser extent JAK2. Baricitinib, with a selectivity to inhibit JAK1 and 2, has been FDA approved for RA in 2018 and by the EMA in 2017. Upadacitinib, a JAK1 selective JAKi, has been approved for RA in 2019 by the FDA and the EMA, followed by filgotinib (JAK1/2 selectivity) in 2020 (EMA). Several other JAKi are currently being examined in clinical trials [5,7].

Many cytokines which are associated with the pathogenesis of RA—such as IL-6, IL-12, IL-15, IL-23, granulocyte-macrophage colony stimulating factor (GM-CSF) and interferons (IFNs)—transduce signals via the JAK-STAT pathway [8,9]. Others, such as IL-1β or TNFα, do not primarily signal via the JAK-STAT pathway, but induce secondary inflammatory loops, e.g., via IL-6 or IFNs, which can be blocked by JAKi [10,11,12,13]. Cytokine receptors preferentially pair with one specific JAK isotype or combination of JAK isotypes. As many of the cytokine receptors signal through a small number of JAKs, inhibition of one JAK isotype will block the signaling of several cytokine receptors or even of a complete cytokine receptor family [6]. This could give JAKi an advantage over bDMARDs which only block one specific cytokine or cytokine receptor, and studies have demonstrated that JAKi are effective in RA patients with inadequate responses to bDMARDs [14,15,16,17].

In the pathogenesis of RA, synovial fibroblasts (SF), also called fibroblast-like synoviocytes, are the main effector cells in joint destruction and perpetuation of inflammation. In the affected joints, SF are the predominant cell type within the characteristic hyperplastic pannus and they develop an aggressive phenotype with tissue-invasive and cartilage as well as bone destructive properties [18,19,20]. RASF secrete large amounts of pro-inflammatory cytokines and promote the infiltration and accumulation of immune cells into the synovial tissues. T lymphocytes, also pivotal actors in RA, constitute about 30–50% of all cell types in the sublining region of the synovial membrane [19,21]. As we and others have shown previously, crosstalk and reciprocal activation between SF and T lymphocytes induce and amplify inflammatory responses and, in particular, interaction with T helper (Th) or B lymphocytes stimulates SF to produce pro-inflammatory cytokines and extracellular matrix degrading enzymes [2,22,23,24,25,26,27,28,29]. On the other hand, the interaction also induces immunosuppressive functions of SF, a capacity that is reduced in RASF [27,28,29,30,31]. Cytokines play a critical role in the interaction between lymphocytes and SF and in the shift towards an aggressive, pro-inflammatory phenotype of SF. However, little is known about how JAKi can influence this bidirectional interplay.

It is well known that JAKi suppress the pro-inflammatory responses of immune cells. Tofacitinib has been shown to significantly inhibit proliferation, cytokine expression, and SF-stimulatory capacity of Th cells [32,33], reduce the T cell-stimulatory capacity of dendritic cells [34] and inhibit the differentiation and antibody production of B cells [14,35]. Synovial biopsies taken from patients treated with tofacitinib have shown decreased expression of matrix metalloproteinases (MMPs) and chemokines such as C-C motif chemokine ligand 2 (CCL2) or C-X-C motif chemokine ligand 10 (CXCL10), whilst the expression of IL-6 or TNFα were not affected [36]. In contrast, treatment of RA synovial explants with tofacitinib in vitro inhibited the production of IL-6, TNFα and other pro-inflammatory cytokines, as well as of MMP1, and decreased the invasive properties of SF [37]. In addition, RASF stimulated with oncostatin M (OSM), IL-17A or TNFα could also be suppressed in their expression of pro-inflammatory cytokines and chemokines by treatment with tofacitinib [10,12,13,37,38]. High concentrations of the panJAKi peficitinib, but not tofacitinib or baricitinib, decreased the IL-1β-induced pro-inflammatory response of RASF [13]. Such data would suggest that the inhibition of JAKs affects the crosstalk between immune cells and SF. However, the specific effects of JAKi on the bidirectional interplay between lymphocytes and SF remain unclear. Therefore, the aim of this study was to investigate the influence of JAKi on the induction of a pro-inflammatory phenotype in SF by activated Th or B cells, on the phenotype of chronically stimulated SF and on the T cell-suppressive capacities of SF. The effects of JAKi were compared to those of established cytokine-neutralizing bDMARDs.

## 2. Materials and Methods

### 2.1. Cell Isolation

SF were isolated from synovial tissues of either osteoarthritis (OA) or RA patients undergoing diagnostic arthroscopy or therapeutic joint surgery as described before [28]. Briefly, synovium samples were digested with collagenase/dispase (Cellsystems, Troisdorf, Germany) for 2 h at 37 °C, passed through cell strainers and cells were cultured in DMEM-F12 medium (Merck, Darmstadt, Germany) containing 10% heat-inactivated fetal calf serum (FCS, Thermo Fisher Scientific, Waltham, MA, USA). Passages 3–9 were used for experiments. Th cells and B cells were freshly isolated from heparinized venous blood by density gradient centrifugation and either MojoSort human CD4 T cell isolation kit (Biolegend, San Diego, CA, USA) for Th cell separation or CD19 Microbeads (Miltenyi Biotec, Bergisch Gladbach, Germany) for B cell isolation. All RA patients fulfilled the classification criteria of the ACR/EULAR [39]. This study was approved by the Ethics Committee of the University of Heidelberg (S-508/2009, 19 Mar 2010).

### 2.2. Cell Culture and Stimulation

T and B lymphocytes were cultured in RPMI-1640 medium (Thermo Fisher Scientific, Waltham, MA, USA) supplemented with 10% FCS. Th cells were stimulated with anti-CD3 and anti-CD28 antibodies (both 1 μg/mL, Biolegend, San Diego, CA, USA). Conditioned culture medium of Th cells (ThCM) was collected on day 4 of culture. B cells were stimulated with Cowan I strain of Staphylococcus aureus (SAC, 1/10,000 (*v*/*v*); Merck, Darmstadt, Germany) and 200 U/mL IL-2 (PeproTech, London, UK) and B cell-conditioned culture media (BcCM) was harvested on day 3.

SF were stimulated with ThCM or BcCM diluted 1:5 in RPMI-1640 medium; for chronic stimulation of SF, ThCM was diluted 1:15. In co-culture experiments, SF were cultured with Th cells in a ratio of 1:5 in RPMI-1640 medium containing 10% FCS and with anti-CD3, anti-CD28 and goat-anti-mouse IgGFab-fragment (6 mg/mL; Jackson ImmunoResearch Laboratories, West Grove, PA, USA).

In experiments targeting JAKs or the IL-6 receptor, SF were pretreated for 1 h with tofacitinib (Pfizer, New York City, NY, USA), baricitinib (TargetMol, Boston, MA, USA), upadacitinib (MedChemExpress, Monmouth Junction, NJ, USA) or tocilizumab (Hoffmann-La Roche, Basel, Switzerland) and then stimulated with ThCM or BcCM for an additional 5 days or co-cultured with Th cells for an additional 6 days. In experiments in which cytokines were targeted by bDMARDs, ThCM or BcCM were pretreated for 1 h with adalimumab (Abbvie, North Chicago, IL, USA), secukinumab or canakinumab (both Novartis, Basel, Switzerland) before the conditioned media-bDMARD mix was added to the SF. BDMARDs were used at final concentrations of 1, 10, or 100 μg/mL. Since no differences were found between these concentrations, only the results of 100 μg/mL bDMARDs are shown here. JAKi were used in concentrations as indicated. Cell viability under treatment was determined by Annexin V and propidium iodide staining and measured by flow cytometry using a BD FACSCanto A analyzer (BD Biosciences, Franklin Lakes, NJ, USA) (results are shown in Figure S1).

### 2.3. Th Cell Proliferation

The cell proliferation was measured by labelling Th cells with the fluorescent cell staining dye carboxyfluorescein succinimidyl ester (CFSE) using the CellTrace CFSE Cell Proliferation Kit (Thermo Fisher Scientific, Waltham, MA, USA) according to the manufacturer’s instructions. The CFSE fluorescence intensity was detected by flow cytometry on day 6 of co-culture (FACSCanto A, BD Biosciences, Franklin Lakes, NJ, USA). Cell divisions were reflected by a reduction in CFSE fluorescence intensity. The suppression of T cell proliferation by synovial fibroblasts was calculated by forming the ratio of the CFSE median fluorescence intensity (MFI) of T cell cultured with SF divided by the CFSE MFI of T cells cultured alone.

### 2.4. Quantification of Cytokine Secretion

The concentrations of IL-6, MMP3, IFNγ, IL-17A and IL-10 in culture supernatants were quantified by enzyme-linked immunosorbent assay (ELISA) using Duo Set ELISA kits (Bio-Techne, Minneapolis, MN, USA) according to the manufacturer’s instructions.

### 2.5. Western Blot Analysis

SF were lysed in RIPA buffer (c.c.pro, Oberdorla, Germany) containing a protease inhibitor cocktail (completeMini, Roche, Basel, Switzerland) for 20 min on ice to obtain total cell protein extracts. Protein samples were diluted in loading buffer, resolved using standard SDS-PAGE and transferred onto PVDF membranes. Indoleamine 2,3-dioxygenase 1 (IDO1) was detected by a rabbit-anti human-IDO antibody (AdipoGen, Liestal, Switzerland). β-Actin was used as a loading control. Densiometric quantification was performed by ImageJ2 Fiji open source software.

### 2.6. Statistics

Statistical significance was analyzed using the Wilcoxon signed-rank test. *p*-values < 0.05 were considered statistically significant. Significance was indicated as follows: *p* **** ≤ 0.0001, *p* *** ≤ 0.001, *p* ** ≤ 0.01, *p* * ≤ 0.05. Results are either presented as grand mean or mean ± SEM.

## 3. Results

### 3.1. JAK Inhibition Dose-Dependently Decreased the Secretion of IL-6 and MMP3 in Co-Cultures of Th Cells and SF

We have previously shown that activated Th cells induce a pro-inflammatory phenotype in co-cultured SF which is characterized by the secretion of large amounts of IL-6 and MMP3 [27]. To investigate the effects of JAKi treatment on this Th cell-induced pro-inflammatory phenotype, we stimulated Th cells in co-culture with either RASF or OASF in the presence or absence of different concentrations of tofacitinib, baricitinib or upadacitinib and analyzed the concentrations of IL-6 and MMP3 in supernatants harvested on day 6 of co-culture.

**Figure 1:**
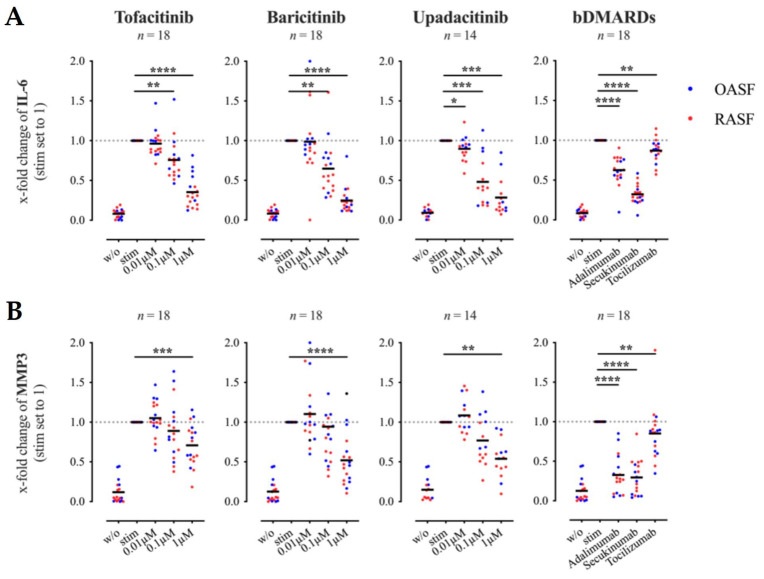
Effects of tofacitinib, baricitinib, upadacitinib and biologic disease modifying anti-rheumatic drugs (bDMARDs) on interleukin (IL)-6 (**A**) and matrix metalloproteinase (MMP)3 (**B**) secretion in SF-Th cell co-cultures. Synovial fibroblasts (SF) from rheumatoid arthritis (RA) patients (RASF in red) or from OA patients (OASF in blue) were co-cultured with Th cells (ratio 1:5) in the presence or absence of anti-CD3/ anti-CD28 antibodies and treated with therapeutics as indicated. The concentration of IL-6 and MMP3 within co-culture supernatants harvested on day 6 was determined by enzyme-linked immunosorbent assay (ELISA). Results are presented as x-fold change with stimulated SF-Th cells set to 1 (mean concentrations ± SEM in co-cultures of SF with stimulated Th cells: IL-6: 600.02 ± 81.47 ng/mL; MMP3: 84.79 ± 22.48 ng/mL). BDMARDs were used at a concentration of 100 μg/mL. Data shown as grand mean, significance tested using Wilcoxon signed-rank test, *p* **** ≤ 0.0001, *p* *** ≤ 0.001, *p* ** ≤ 0.01, *p* * ≤ 0.05.

The secretion of IL-6 was suppressed in a dose-dependent manner by all three JAKi tested (Figure 1A). At a concentration of 0.01 μM, only upadacitinib was able to affect the IL-6 production, whilst all tested JAKi attenuated the secretion of IL-6 significantly at a concentration of 0.1 μM and which was further enhanced at 1 μM (Figure 1A). MMP3 release was also significantly reduced by the JAKi tested at a concentration of 1 μM (Figure 1B). As there was no significant difference between results obtained with RASF or OASF, the results of both SF were combined.

The inhibition of JAK-STAT signaling by JAKi can affect signal transduction of several different cytokine receptors simultaneously, JAKi may be more effective than bDMARDs in inhibiting SF activation by Th cells. To prove this hypothesis, we analyzed the effects of adalimumab (anti-TNFα), secukinumab (anti-IL-17A) or tocilizumab (anti-IL-6 receptor) on IL-6 and MMP3 production by SF co-cultured with activated Th cells. Remarkably, all tested bDMARDs significantly decreased the secretion of IL-6 and MMP3 (Figure 1A,B). However, the effect of tocilizumab on IL-6 and MMP3 expression was very weak. Secukinumab suppressed the release of IL-6 best, comparable to the effects of JAKi at a concentration of 1 μM (Figure 1A). Both secukinumab and adalimumab strongly attenuated the secretion of MMP3 by SF (Figure 1B). Thus, JAKi were not superior to the bDMARDs secukinumab or adalimumab in blocking the Th cell-mediated induction of a pro-inflammatory phenotype in SF.

**Figure 2:**
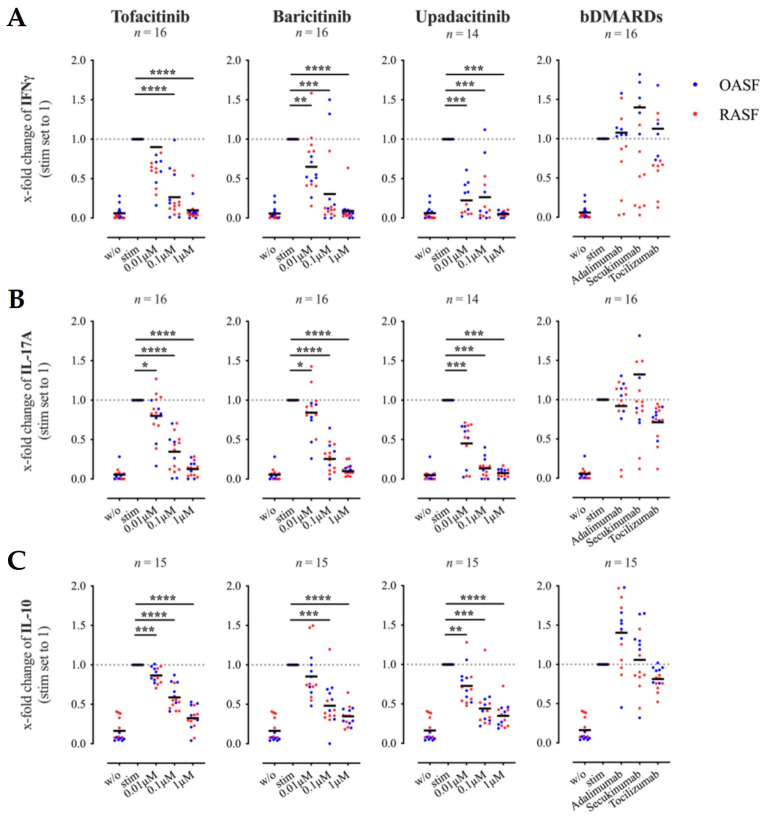
Effects of tofacitinib, baricitinib, upadacitinib and bDMARDs on interferon (IFN)γ (**A**), IL-17A (**B**) and IL-10 (**C**) secretion in Th cell-SF co-cultures. RASF (red) or OASF (blue) were co-cultured with Th cells (ratio 1:5) in the presence or absence of anti-CD3/ anti-CD28 antibodies and drugs as indicated. Results are presented as x-fold change with stimulated SF-Th cells set to 1 (mean concentrations ± SEM in co-cultures of SF with stimulated Th cells (in pg/mL): IFNγ: 452.06 ± 137.67; IL-17A: 6772.01 ± 1689.89; IL-10: 2140.26 ± 185.16). Supernatants were collected on day 6 and cytokine concentrations were quantified by ELISA. Data shown as grand mean, significance tested using Wilcoxon signed-rank test, *p* **** ≤ 0.0001, *p* *** ≤ 0.001, *p* ** ≤ 0.01, *p* * ≤ 0.05.

Cytokines play a key role in crosstalk between Th cells and SF. Therefore, we analyzed the effects of JAKi on cytokine expression by activated Th cells in the same experimental setting. Secretion of IFNγ, IL-17A, and IL-10 in Th cell-SF co-cultures were greatly reduced by treatment with tofacitinib, baricitinib or upadacitinib (Figure 2A–C). All JAKi tested significantly decreased the release of IL-17A already at a concentration of 0.01 μM, while only upadacitinib and baricitinib significantly reduced the release of IFNγ at a concentration of 0.01 μM. A concentration of 1 μM JAKi decreased IFNγ and IL-17A secretion almost to the levels of unstimulated Th cells. (Figure 2A,B). However, not only the secretion of potentially pro-inflammatory T cell-cytokines was suppressed by JAKi; the production of the immunosuppressive cytokine IL-10 was significantly and dose-dependently decreased by all of the JAKi tested as well. In contrast to their effectiveness on IL-6 and MMP3 secretion, adalimumab or secukinumab had no effect on the release of IFNγ, IL-17A or IL-10 in Th cell-SF co-cultures. Only tocilizumab slightly attenuated IL-17A and IL-10, but not IFNγ secretion (Figure 2A–C). We also analyzed the effects of JAKi on cytokine expression of Th cells cultured alone. Tofacitinib, baricitinib and upadacitinib dose-dependently suppressed the secretion of IFNγ, IL-17A and IL-10 by Th cells stimulated in mono-culture (Figure S2). At a concentration of 0.01 μM, all of the JAKi tested reduced the cytokine production significantly; concentrations of 1 μM lowered the cytokine levels to those found in cultures of unstimulated Th cells.

Taken together, tofacitinib, baricitinib and upadacitinib suppressed cytokine secretion by SF as well as Th cells in co-cultures, while adalimumab and secukinumab affected the pro-inflammatory phenotype of SF induced by activated Th cells but not the cytokine expression of the Th cells themselves.

### 3.2. JAKi Directly Suppressed the Secretion of IL-6 and MMP3 by SF Stimulated with Soluble Factors Released by Th Cells

The suppressive effects of JAKi on IL-6 and MMP3 expression by SF co-cultured with Th cells could be due to a suppression of cytokine secretion of Th cells, which would then consequently lead to decreased SF activation in co-cultures. Alternatively, they could also be due to a direct inhibition of signal transductions in SF. In order to test whether JAKi have the capacity to directly suppress pro-inflammatory responses of SF, we stimulated SF with ThCM in the presence or absence of different concentrations of tofacitinib, baricitinib or upadacitinib and analyzed the secretion of IL-6 and MMP3 by SF on day 5 of culture.

**Figure 3:**
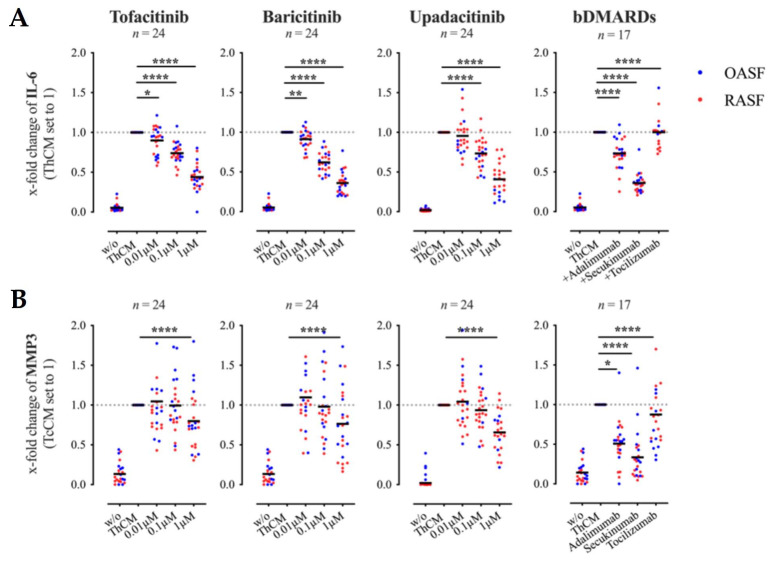
Effects of tofacitinib, baricitinib, upadacitinib and bDMARDs on IL-6 (**A**) and MMP3 (**B**) expression by SF stimulated with conditioned culture medium of Th cells (ThCM). Th cells were stimulated with anti-CD3/anti-CD28 antibodies and supernatants (ThCM) were collected on day 4. RASF (red) or OASF (blue) were cultured with or without ThCM and treated, respectively. Supernatants were collected on day 5 and analyzed by ELISA. Results are presented as x-fold change with SF stimulated with ThCM set to 1 (mean concentrations ± SEM in ng/mL: IL-6: 244.64 ± 20.62; MMP3: 42.64 ± 8.97). Data shown as grand mean, significance tested using Wilcoxon signed-rank test, *p* **** ≤ 0.0001, *p* ** ≤ 0.01, *p* * ≤ 0.05.

All JAKi dose-dependently decreased the secretion of IL-6 by SF (Figure 3A). A significant reduction in IL-6 production was achieved with tofacitinib and baricitinib at a concentration of 0.01 μM and with upadacitinib at a concentration of 0.1 μM (Figure 3A). In contrast, significant inhibition of MMP3 secretion was only detected in cultures which were treated with the highest concentration of 1 μM (Figure 3B). Analogous to the co-culture experiments, the effects of JAKi on IL-6 and MMP3 expression by SF were compared with those of the bDMARDs adalimumab, secukinumab or tocilizumab. Similar to the results seen in SF-Th cell co-cultures, secukinumab reduced the secretion of IL-6 by ThCM-stimulated SF by more than 50%, comparable to the effects of 1μM JAKi. Treatment with adalimumab also significantly reduced IL-6 production by SF, albeit to a lesser extent than secukinumab. Both adalimumab and, even more so, secukinumab strongly inhibited the secretion of MMP3 by the SF (Figure 3B). Tocilizumab had no effect on IL-6 nor on MMP3 expression by SF stimulated with ThCM.

**Figure 4:**
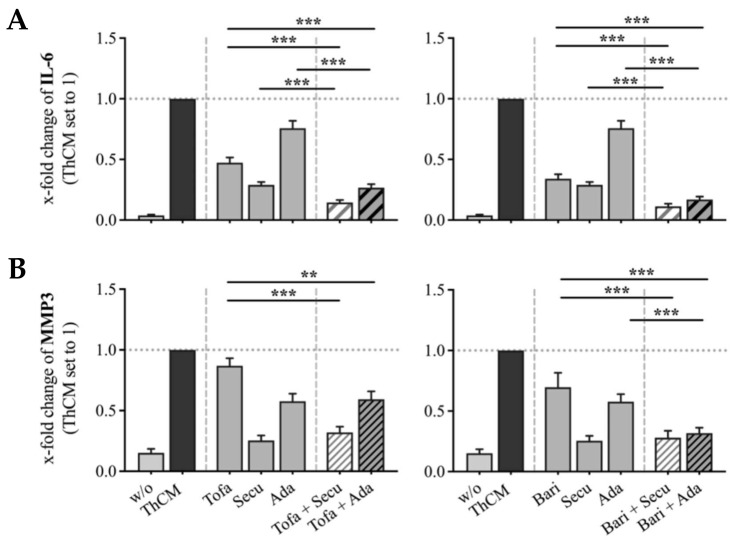
Individual and combined effects of tofacitinib, baricitinib and bDMARDs on IL-6 (**A**) and MMP3 (**B**) secretion by SF stimulated with ThCM. SF (OASF (*n* = 6), RASF (*n* = 6)) were cultured in the presence or absence of ThCM and treated with JAKi or bDMARDs individually or in combinations as indicated. Supernatants were collected on day 5 and IL-6 and MMP3 quantified by ELISA. Results are presented as x-fold change with SF stimulated with ThCM set to 1. Concentrations used: tofacitinib 1μM, baricitinib 1μM with either secukinumab 100 μg/mL or adalimumab 100 μg/mL. *n* = 12. Data shown as mean with SEM, significance tested using Wilcoxon signed-rank test, *p* *** ≤ 0.001, *p* ** ≤ 0.01.

Next, we tested whether a combination of JAKi and bDMARDs would have a greater effect on IL-6 or MMP3 expression by ThCM-stimulated SF as compared to the individual inhibitory effects. Therefore, SF were stimulated with ThCM in the absence or presence of tofacitinib, baricitinib, adalimumab or secukinumab alone or with a combination of a JAKi with either of the bDMARDs. Treatment with tofacitinib or baricitinib in combination with either adalimumab or secukinumab, significantly reduced the secretion of IL-6 by SF as compared to SF treated with one individual JAKi or bDMARD (Figure 4A). However, MMP3 secretion mediated by secukinumab was not further decreased by simultaneous treatment with JAKi (Figure 4B). Only a combined treatment with baricitinib and adalimumab resulted in a significantly stronger inhibition of MMP3 production by SF compared to the individual inhibitory effects (Figure 4B).

These results demonstrate that the suppressive effects of JAKi are not only due to a reduction in Th cell cytokine expression, but also caused by a direct blocking of signal transductions in SF. Furthermore, certain combined treatments with JAKi and bDMARDs achieved even greater suppressive effects on IL-6 and MMP3 expression in ThCM-stimulated SF compared to individual effects.

### 3.3. JAKi Decreased the Secretion of IL-6 by SF Stimulated with Soluble Factors Released by B Cells, but Were Ineffective in Inhibiting the Secretion of MMP3

**Figure 5:**
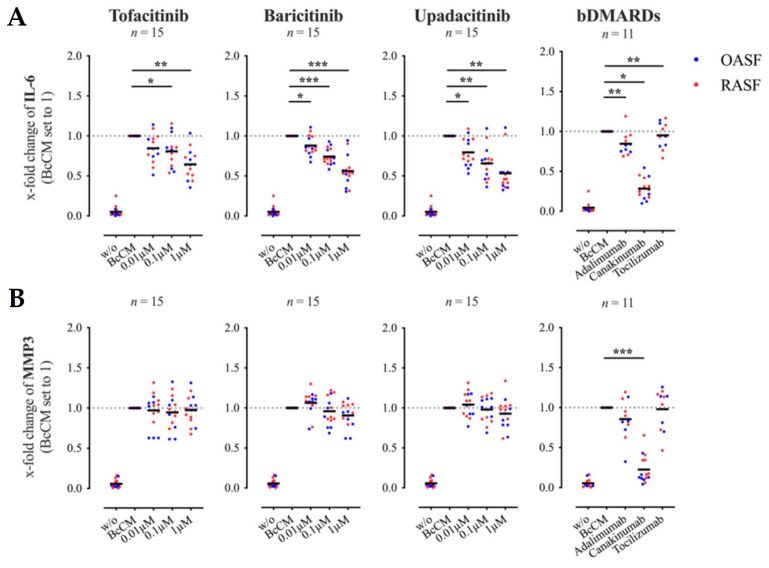
Effects of tofacitinib, baricitinib, upadacitinib and bDMARDs on IL-6 (**A**) and MMP3 (**B**) secretion by SF stimulated with B cell-conditioned culture media (BcCM). B cells were stimulated with staphylococcus aureus Cowan I (SAC) plus IL-2 and supernatant (BcCM) was collected on day 3. OASF (blue) and RASF (red) were cultured with or without BcCM and drugs as indicated for 5 days. Supernatant was collected and cytokines quantified by ELISA. Results are presented as x-fold change with SF stimulated with BcCM set to 1 (mean concentrations ± SEM in ng/mL: IL-6: 215.21 ± 42.02; MMP3: 244.95 ± 49.37). bDMARDs shown were used at a concentration of 100 μg/mL. Data shown as grand mean, significance tested using Wilcoxon signed-rank test, *p* *** ≤ 0.001, *p* ** ≤ 0.01, *p* * ≤ 0.05.

Similar to Th cells, activated B cells release soluble factors that induce an inflammatory phenotype in SF with increased production of IL-6 and MMPs [29]. However, the composition of cytokines released by B and Th cells are different. In the crosstalk between SF and Th cells, cytokines such as IL-17A, IFNγ and TNFα play major pro-inflammatory roles, whilst TNFα and IL-1β have been shown to be key in the interplay between SF and B cells. To investigate the effects of JAKi on the B cell-induced pro-inflammatory phenotype, SF were stimulated with BcCM in the presence or absence of different concentrations of the JAKi tofacitinib, baricitinib or upadacitinib. In parallel, the inhibitory capacities of adalimumab, tocilizumab and canakinumab (anti-IL-1β) on B cell-stimulated SF were tested. All JAKi significantly and dose-dependently decreased the secretion of IL-6 by SF stimulated with BcCM (Figure 5A). Treatment with canakinumab strongly inhibited the production of IL-6, adalimumab had a slight but significant suppressive effect, while tocilizumab had no effect on IL-6 secretion (Figure 5A). Contrary to their effects on the secretion of IL-6, none of the JAKi tested showed an effect on the expression of MMP3 by SF stimulated with BcCM (Figure 5B). Only treatment with canakinumab significantly reduced MMP3 secretion by SF.

Thus, unlike the significant reduction in MMP3 in ThCM stimulated SF, JAKi had no effect on MMP3 expression in BcCM stimulated SF. The strongest inhibition on IL-6 and MMP3 secretion was achieved by treatment with the bDMARD canakinumab.

### 3.4. Even in Chronically Stimulated SF, Inhibition of JAKs Suppressed IL-6 and MMP3 Secretion

In a previous study, we showed that chronic stimulation of SF by soluble factors released by activated Th cells resulted in a significantly enhanced inflammatory response as compared to a single or a second stimulation [40]. This might be important as RA treatment with these targeted drugs is normally initiated when arthritis is already ongoing for months to years. To test the capacity of tofacitinib or adalimumab to suppress the inflammatory memory response of chronically stimulated SF, OASF were repeatedly stimulated by ThCM for a total period of 16 days, then washed and left unstimulated for two more days. On day 18, OASF were either (i) left unstimulated, (ii) re-stimulated by ThCM, (iii) re-stimulated by ThCM in the presence of tofacitinib (1 μM) or (iv) re-stimulated by ThCM in the presence of adalimumab (100 μg/mL). On day 22, culture supernatants were harvested and IL-6 and MMP3 levels were quantified. In parallel, OASF were cultured for 16 days under resting conditions and, starting from day 18, were treated analogously to the chronically stimulated SF (primary stimulated SF).

**Figure 6:**
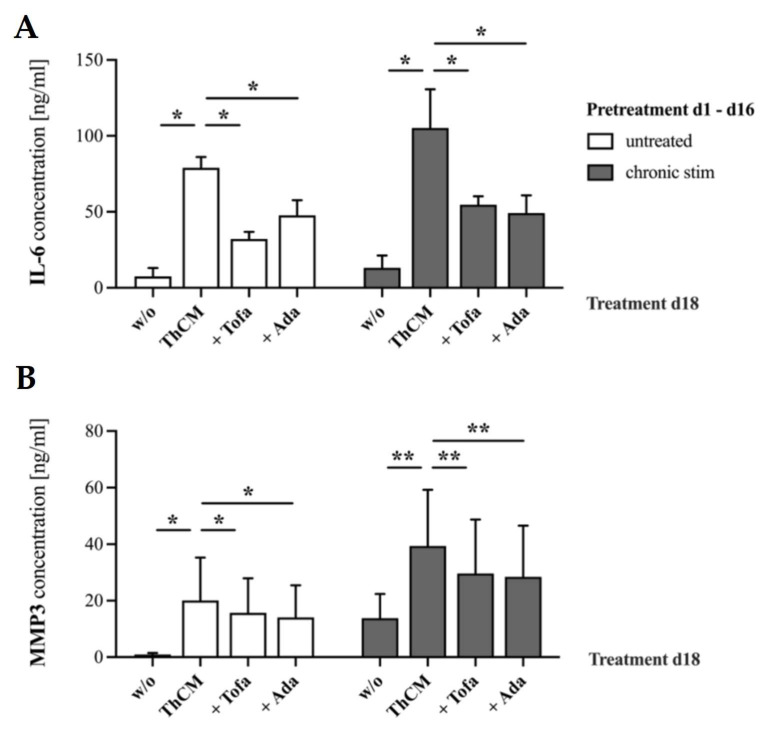
Effects of tofacitinib and adalimumab on IL-6 (**A**) and MMP3 (**B**) expression by chronically stimulated compared to previously unstimulated SF. OASF were either left untreated or were continuously restimulated with ThCM for 16 days, then washed and left unstimulated for two more days. On day 18, SF were either (i) left unstimulated (w/o), (ii) re-stimulated by ThCM, (iii) re-stimulated by ThCM in the presence of tofacitinib (1 μM) or (iv) re-stimulated by ThCM in the presence of adalimumab (100 μg/mL). On day 22, supernatant was collected and IL-6 and MMP3 concentrations were quantified by ELISA. Data shown as mean ± SEM, significance tested using Wilcoxon signed-rank test, *p* ** ≤ 0.01, *p* * ≤ 0.05. *n* = 6 for IL-6, *n* = 8 for MMP3.

Stimulation on day 18 by ThCM significantly increased the secretion of IL-6 and MMP3 by primary stimulated SF as well as chronically stimulated SF (Figure 6). Treatment with either tofacitinib or adalimumab significantly reduced the secretion of IL-6 and MMP3 by primary stimulated SF, confirming the previous results (Figure 3). Importantly, the inhibition of JAKs by tofacitinib significantly suppressed IL-6 (Figure 6A) and MMP3 (Figure 6B) production even in chronically stimulated SF. Similarly, treatment with adalimumab efficiently reduced the secretion of IL-6 and MMP3 by SF which had previously been chronically stimulated with ThCM (Figure 6A,B).

Thus, the inhibition of JAKs or the neutralization of TNFα not only attenuated the inflammatory response of primary stimulated SF, but also significantly suppressed the enhanced memory response of chronically stimulated SF.

### 3.5. JAKi, but Not bDMARDs, Reduced the IDO1-Mediated Suppression of T Cell-Proliferation by SF

Bidirectional crosstalk between Th cells and SF leads not only to the induction of a pro-inflammatory phenotype of SF, but also to the suppression of T cell responses by SF. As we have shown previously, SF stimulated by IFNγ possess the capacity to suppress the proliferation of co-cultured Th cells through IDO1-mediated tryptophan metabolism [27]. This negative feedback mechanism of suppression is thought to participate in the prevention of excessive Th cell responses. The efficacy of JAKi in RA treatment could suggest that JAKi may support the immunosuppressive capacities of SF. In order to prove this hypothesis, Th cells were labeled with the fluorescent cell staining dye CFSE and stimulated in mono-culture or in co-culture with SF in the presence or absence of different concentrations of tofacitinib, Baricitinib, upadacitinib or bDMARDs.

**Figure 7:**
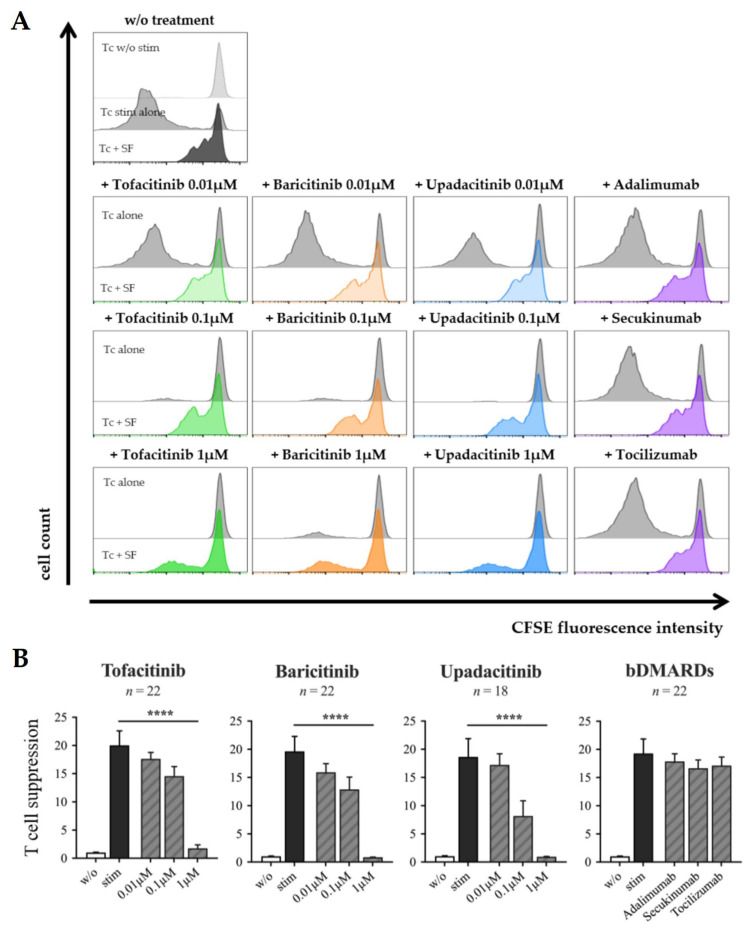
Effects of tofacitinib, baricitinib, upadacitinib and bDMARDs on the suppression of Th cell-proliferation by SF. CFSE-labeled Th cells were cultured alone or in co-culture with SF (OASF (*n* = 10–12), RASF (*n* = 8–10)) in the presence or absence of anti-CD3/ anti-CD28 and drugs as indicated. Cells were harvested on day 6 and T cell-proliferation was determined by flow cytometry. (**A**) Histograms from one representative experiment. (**B**) Suppression of Th cell-proliferation as detected in all experiments. T cell suppression was calculated by dividing the CFSE median fluorescence intensity (MFI) of Th cells co-cultured with SF by those of Th cells cultured alone. Data shown as mean ± SEM, significance tested using Wilcoxon signed-rank test, *p* **** ≤ 0.0001.

In co-cultures, Th cell proliferation was strongly suppressed by SF, confirming previous results (Figure 7A,B). Treatment of co-cultures with JAKi dose-dependently attenuated the capacity of SF to suppress the proliferation of Th cells. At a concentration of 1μM, all of the JAKi tested significantly reduced the suppressive capacities of SF (Figure 7B). In contrast, the addition of the bDMARDs adalimumab, secukinumab or tocilizumab to co-cultures of Th cells and SF had no effect on the SF-mediated suppression of Th cell proliferation (Figure 7A,B).

**Figure 8:**
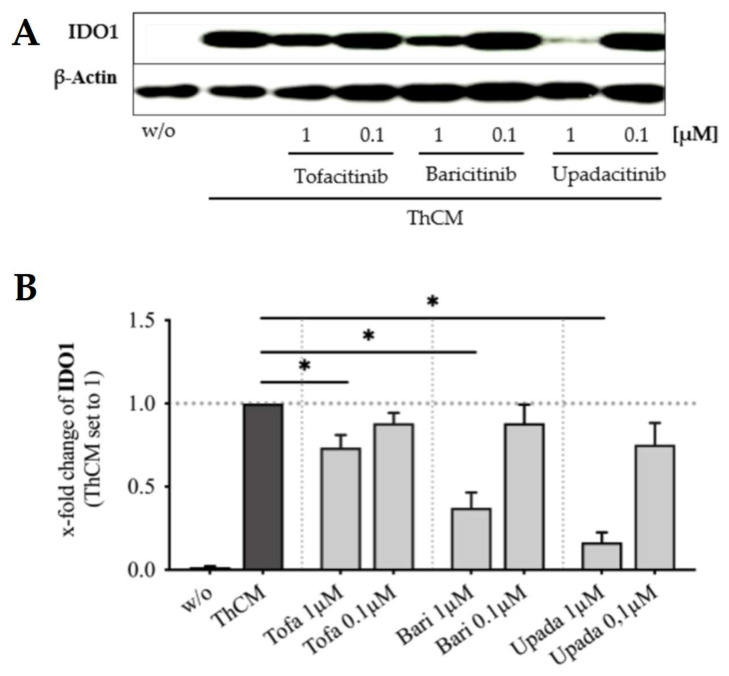
Effects of tofacitinib, baricitinib and upadacitinib on the expression of IDO1 by SF stimulated with ThCM. OASF or RASF were left untreated (w/o) or stimulated with ThCM and treated with tofacitinib (*n* = 7), baricitinib (*n* = 7) or upadacitinib (*n* = 5). On day 4, SF were harvested and whole cell extracts were subjected to immunoblot analysis. Shown are the results from one representative experiment (**A**) and the x-fold change of indoleamine 2,3-dioxygenase 1 (IDO1) relative to b-actin expression with SF stimulated with ThCM set as 1 detected in all experiments (**B**). Data shown as mean ± SEM, significance tested using Wilcoxon signed-rank test, *p* * ≤ 0.05.

As shown in our previous study, tryptophan catabolism mediated by IDO1 expression in SF is responsible for suppressing the proliferation of Th cells [27]. Therefore, we examined the effects of JAK inhibition on the expression of IDO1 by SF stimulated with ThCM. Treatment of SF with 1μM tofacitinib, baricitinib or upadacitinib significantly suppressed the ThCM-induced expression of IDO1 (Figure 8A,B). Upadacitinib caused the largest reduction in IDO1 expression by SF, and tofacitinib the smallest. Treatment with adalimumab, secukinumab, or tocilizumab had no effect on IDO1 expression by SF stimulated with ThCM (Figure S3).

Thus, the assumption that JAKi may support the immunosuppressive capacities of SF was not confirmed by these results. Instead, JAKi, but not bDMARDs, attenuated the IDO1-mediated suppression of Th cell proliferation by SF.

## 4. Discussion

Crosstalk between SF and immune cells plays a central role in the pathogenesis, chronicity, and destructive nature of RA. In RA synovium, the released cytokines are key drivers for the vicious, pro-inflammatory cycle of the SF-immune cell interaction. JAKi represent a promising treatment option, since the inhibition of JAKs results in the suppression of signaling of multiple cytokine receptors simultaneously. Exposure of SF to synovial fluid of RA patients has been shown to activate the JAK-STAT signaling pathway [41,42] and the receptors of many cytokines and chemokines that play important roles in the pathogenesis of RA—such as IL-6, RANTES, MCP-1, IP-10, OSM, and IFNs—directly transmit signals via the JAK-STAT pathway [43]. TNFα, although not directly associated with the JAK-STAT pathway, induces a delayed, secondary activation of JAK-STAT signaling in SF [10,12]. In previous studies, the suppressive effects of JAKi on SF stimulated by one of these cytokines has been examined. In SF stimulated with OSM, tofacitinib and baricitinib were found to similarly inhibit the phosphorylation of JAKs and STATs and to suppress the secretion of IL-6 and MCP-1 [13,37,38,44]. Both JAKi also diminished TNF-induced interferon-signals and associated inflammatory responses in SF [10,12,45]. In IL-1β-stimulated SF, high concentrations (5 μM) of peficitinib, but not of tofacitinib or baricitinib, decreased the release of IL-6, MMP3, CXCL1 and CXCL8 [13]. These studies clearly demonstrated the efficacy of JAKi in suppressing inflammatory responses in cytokine-stimulated SF.

**Figure 9:**
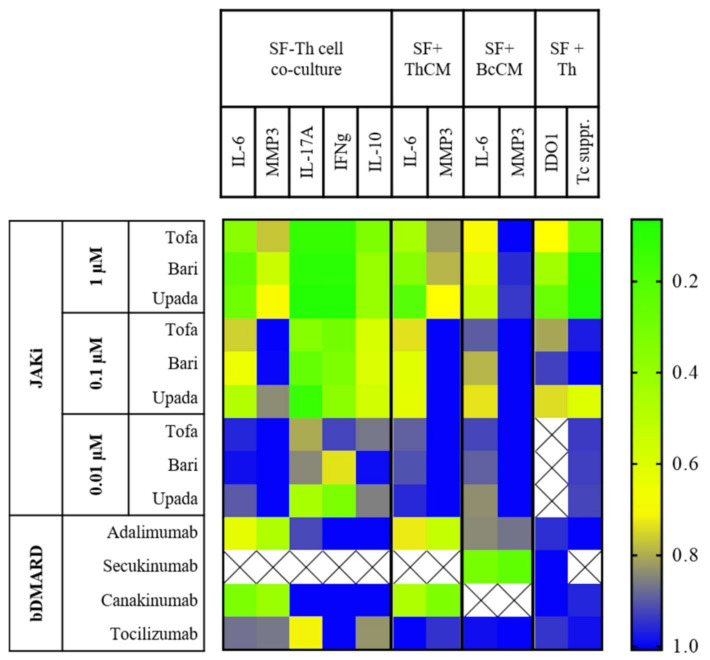
Schematic summary of the effects of JAKi and bDMARDs on the SF or Th cell phenotype. The figure summarizes the results of this study by presenting the results as a heatmap. A value of 1.0 (blue) means no suppressive effect of JAKi or bDMARDs, a value of 0.0 (green) a 100% reduction in the corresponding cell function compared to untreated cells.

In this study, we focused on the effects of JAK inhibition on the crosstalk between immune cells and SF and, in particular, on the induction of an aggressive, pro-inflammatory phenotype in SF by lymphocytes. We analyzed the effects of the pan-JAKi tofacitinib, the moderately selective JAK1 and JAK2 inhibitor baricitinib and the selective JAK1 inhibitor upadacitinib on the crosstalk between SF and lymphocytes and compared them with those of bDMARDs. All experiments and results of our study are summarized in Figure 9. We show that all tested JAKi significantly suppressed the secretion of IL-6 and MMP3 as well as of IFNγ, IL-17A and IL-10 in SF and Th cell co-cultures. The effectiveness of JAKi in suppressing IL-6 and MMP3 secretion even in ThCM-stimulated SF demonstrated that these suppressive effects of JAKi are not only due to a suppression of cytokine secretion by Th cells, which would in turn attenuate the induction of a pro-inflammatory phenotype in SF, but also to a direct inhibition of JAK-STAT signaling in SF.

A significant reduction in IL-6 secretion was already achieved at a concentration of 0.01–0.1 μM tofacitinib, baricitinib or upadacitinib. However, the secretion of MMP3 was only significantly suppressed at 1 μM of all JAKi tested in SF stimulated by co-cultured Th cells as well as in ThCM-stimulated SF. Of note, the plasma Cmax levels of all three JAKi in patients under medication with approved doses are around 0.1 μM and thus well below 1 μM [46,47,48]. B cells activate SF by a different set of cytokines than Th cells. Nevertheless, the secretion of IL-6 from SF stimulated with soluble factors released by B cells was significantly reduced by JAKi at a concentration of 0.1 μM, similar to the effects on ThCM-stimulated SF. Baricitinib and upadacitinib inhibited the IL-6 secretion of BcCM-stimulated SF even at a concentration of 0.01 μM. On the other hand, MMP3 expression of SF stimulated by BcCM could not be suppressed by the JAKi tested, not even at a concentration of 1 μM. These data would indicate that the stimulation of IL-6 expression by SF—no matter if stimulated by Th or B cells—is more dependent on JAK-STAT signaling than that of MMP3. In accordance with that, Boor et al. showed that tofacitinib treatment of SF stimulated by IFNα and TLR3 ligation only significantly suppressed IL-6, but not MMP3 expression [49].

A potent suppression of IL-6 and MMP3 secretion could be achieved by treating ThCM-stimulated SF with adalimumab or secukinumab, and by treating BcCM-stimulated SF with canakinumab. These findings emphasize the pivotal roles of TNFα and IL-17A—released by T cells—and IL-1β—released by B cells—in the induction of a pro-inflammatory, matrix-degrading phenotype in SF. This is consistent with the previously described functions of these cytokines in the vicious cycle of SF–immune cell interaction in RA [19]. Considering that TNFα, IL-17A and IL-1β are strong inducers of IL-6 and MMP3 expression by SF, it is noteworthy that the receptors for these three cytokines do not directly transmit signals via the JAK-STAT pathway. Nevertheless, inhibition of JAKs has been demonstrated to suppress pro-inflammatory responses of SF stimulated by TNFα, IL-17A or IL-1β [10,12,13,37]. TNFα-stimulation of SF rapidly activates MAPK and NFκB signaling pathways [50], but it also stimulates the upregulation of IRF1 in SF, which in turn induces the expression of IFNβ. IFNβ activates the JAK-STAT pathway and the expression of IFN-response genes. Both could be blocked by tofacitinib and baricitinib [10,12]. Furthermore, the effectiveness of tofacitinib on the suppression of IL-17A induced IL-6 expression has already been shown by McGarry et al. [37]. IFNγ is another T cell-cytokine well-known to induce an aggressive phenotype in SF, whose receptor signaling can be blocked by JAKi. As shown in this study, the secretion of IFNγ itself is already strongly suppressed by JAKi treatment in Th cell mono-cultures as well as in SF-Th cell co-cultures. Furthermore, baricitinib treatment was shown to significantly diminish the invasive behavior of IFNγ-stimulated SF [51].

In this study, we have shown that both JAKi and neutralization of TNFα suppressed the expression of IL-6 and MMP3 by Th cell-stimulated SF. Importantly, treatment of ThCM-stimulated SF with a combination of adalimumab and tofacitinib or baricitinib reduced the IL-6 secretion significantly more than adalimumab or one individual JAKi alone. The combined treatment with adalimumab and baricitinib, but not tofacitinib, also resulted in significantly stronger inhibition of MMP3 secretion by SF as compared to the individual inhibitory effects. This indicates that TNF-stimulation additionally activates JAK-STAT-independent signaling pathways that support IL-6 and MMP3 expression by SF which cannot be blocked by JAKi alone. Similar to adalimumab, a combined treatment of Th cell-stimulated SF with secukinumab and tofacitinib or baricitinib led to a significantly stronger inhibition of IL-6 secretion as compared to the individual effects. However, the suppression of MMP3 expression by secukinumab was not further enhanced by the JAKi. Such data again highlights the complexity of a multi-level inflammatory network. In the case of stimulation of SF by B cell-released factors, canakinumab strongly suppressed the release of both IL-6 and MMP3, while JAK inhibition only decreased IL-6, but not MMP3 production. Thus—similar to TNFα—IL-17A and IL-1β activated signaling pathways that induce IL-6 and MMP3 secretion by SF which cannot be blocked by JAKi. Clinically, such inefficient suppression of TNFα, IL-17A or IL-1β signaling in SF could result in limited responses to JAKi treatments in RA patients. A combination of a JAKi with a bDMARD, as shown here, might be an option in the treatment of individual patients. Moreover, it has been shown that cytokine-neutralizing bDMARDs, which are ineffective in one rheumatic disease, can still work convincingly in another. For example, TNFα-, IL-6R- and IL-1β-neutralizing bDMARDs work in RA, whereas IL-17A and IL-12/23-neutralizing bDMARDs are very efficient in psoriatic arthritis or spondyloarthritis. JAKi seem to work in most of the mentioned rheumatic diseases, but not in every patient with similar efficacy. A combination of two different cytokine-neutralizing bDMARDs did not yield a superior effect as shown in several clinical trials, but appeared to increase the risk of serious side effects [52,53,54]. According to observations and the data presented in this study, a combination of a JAKi with a cytokine-neutralizing bDMARD could provide a more effective treatment strategy. However, the clinical safety and efficacy of such a strategy would need to be established [55].

We could show that JAK inhibition significantly inhibited the secretion of IL-6 and MMP3 even in chronically stimulated SF. The pathogenesis of RA is characterized by chronic, persistent inflammation and SF are known to play a central role in the switch from acute resolving to chronic persistent inflammation [20,56]. An inflammatory microenvironment not only induces a shift in SF phenotype towards inflammation and cartilage and bone destruction, but also leads to the imprinting of this aggressive phenotype, attributed at least in part to epigenetic modifications [20,57]. Remarkably, a pro-inflammatory memory has been described for SF primed by an inflammatory stimulus [58], and chronic stimulation of SF by ThCM triggered a significantly enhanced inflammatory memory response as compared to a single or even a second stimulation [40]. Hence, to be effective, any therapeutic option should be able to suppress the pro-inflammatory memory response of chronically activated SF. Our data demonstrates that tofacitinib as well as adalimumab significantly suppressed the release of IL-6 and MMP3 not only by primary stimulated SF, but also by chronically stimulated SF with ThCM. Furthermore, we have previously shown that chronic stimulation of SF not only resulted in the development of an “inflammatory memory”, but also in that of a “glycolytic memory”. On top of an enhanced secretion of IL-6 and MMP3, repeated stimulation of SF with ThCM triggered a significantly increased glycolytic activity as compared to unstimulated, singly stimulated or even restimulated SF [40]. A dysregulated glucose metabolism in SF has been suggested to play a critical role in the pathogenesis of RA [59] and tofacitinib has been shown to suppress the glycolytic activity of RASF [37]. Further investigation is required to verify whether JAKi also inhibit the glycolytic memory response of chronically stimulated SF.

As shown by our group and others, SF bear the capacity to efficiently suppress T and B cell responses [27,29,30,31]. Therapeutic strategies for RA could potentially be improved if they modulated SF in a way that could suppress its pro-inflammatory, aggressive properties, while at the same time promoting its anti-inflammatory, immunosuppressive functions. Both immunosuppressive and inflammatory properties of SF are induced by lymphocytes in a cell contact-independent manner, suggesting that cytokines and corresponding signaling pathways are involved. The efficacy of JAKi in RA treatment implied that it is possible to shift the SF phenotype towards an immunosuppressive, anti-inflammatory phenotype. However, as shown here, tofacitinib, baricitinib and upadacitinib strongly decreased the suppressive effects of SF on Th cell proliferation and also significantly suppressed the expression of IDO1 and IL-10. The immunosuppressive cytokine IL-10 is known to counterbalance the chronic activation of innate and adaptive immune cells in RA and IL-10 producing cell types such as regulatory T cells (Treg), but also regulatory B cells (Breg), macrophages or dendritic cells (DCs), play an important role in the resolution of inflammation [60]. In mouse models of RA, administration of neutralizing anti-IL-10 antibodies showed an acceleration of arthritis [61], while intraarticular overexpression of IL-10 diminished synovitis and cartilage proteoglycan depletion [62]. Interestingly, while the secretion of IL-10 by Th cells was strongly suppressed by JAKi, as shown here, other studies showed no attenuation of its secretion by other cells. Although tofacitinib inhibited differentiation and antibody production by B cells, the production of IL-10 was not affected [35]. In DCs, the IL-10 production was even increased by tofacitinib treatment [34].

As shown previously, IDO1-activity is essential for the T cell-suppressive capacity of SF, since blocking of IDO1 activity completely restored Th cell proliferation in Th cell-SF co-cultures [27]. In addition, IDO1 expression is induced in SF by IFNγ secreted by activated Th cells and blocking IFNγ inhibited the IDO1-mediated tryptophan catabolism by ThCM-stimulated SF [27]. The IFNγ receptor is associated with JAK1 and JAK2 and, therefore, tofacitinib, baricitinib and upadacitinib should inhibit the signal transduction of the IFNγ receptor. Moreover, as shown here, all JAKi significantly suppressed the secretion of IFNγ in SF-Th cell co-cultures. Thus, the JAKi-related reduction in IDO1 expression and of the suppression of T cell-proliferation by SF, could be due to a suppression of both IFNγ secretion and IFNγ receptor signaling by the JAKi. Interestingly, upadacitinib most efficiently decreased IDO1 expression in ThCM-stimulated SF and also most strongly reduced IFNγ secretion by Th cells even at a concentration of 0.01 μM. Compared with that, tofacitinib showed the lowest suppression of IDO1 and also the least effects on IFNγ secretion in Th cell-SF cultures. In contrast to the JAKi tested, neither adalimumab, secukinumab nor tocilizumab attenuated IDO1 expression, IFNγ secretion or T cell-suppressive effects of SF. The reversal of the immunosuppressive capacities of SF through JAK inhibition could be a disadvantage of JAKi therapy. Yet, a significant reduction in both IDO1 expression and suppression of Th cell proliferation was only achieved at a concentration of 1 μM of the JAKi tested. At a concentration of 0.1 μM, which corresponds more closely to the plasma Cmax levels achieved with approved JAKi doses, none of the JAKi tested significantly decreased IDO1 expression or Th cell-suppression by SF. Furthermore, since the inhibition of JAKs itself already strongly inhibits T cell-proliferation [33] and cytokine expression, a weakening of the T cell-suppressive functions of SF by JAKi would not necessarily lead to an overbalance of inflammation. Moreover, whilst the inhibition of JAKs might reduce the immunosuppressive capacities of SF, it could at the same time enhance those of other cells. For instance, tofacitinib has been described to increase the expression of IDO1, IDO2 and IL-10 in lipopolysaccharide-stimulated dendritic cells and to decrease their T cell-stimulatory capacities [34,63], again highlighting the enormous complexity of the humoral and cellular network of the human immune system.

In conclusion, JAKi were able to limit pro-inflammatory circuits in the crosstalk between Th cells and SF by significantly suppressing the cytokine production of Th cells and the IL-6 and MMP3 secretion of SF stimulated by factors released by Th cells. Importantly, even in chronically stimulated SF, the secretion of IL-6 and MMP3 could be suppressed by JAKi. However, JAKi treatment not only diminished the pro-inflammatory, but also the immunosuppressive properties of SF. Both of these effects—the suppression of pro-inflammatory and of immunosuppressive circuits in the interplay between SF and lymphocytes—were strongly dependent on the JAKi dose. The resulting differences between IL-6, MMP3 and IDO1 expression by SF in their response to JAKi doses may contribute to the heterogeneity of clinical responses to JAKi treatment in RA patients. Finally, although JAKi have been supposed to be superior to bDMARDs as they target the signaling of multiple cytokine receptors simultaneously, certain bDMARDs were at least as efficient as JAKi in suppressing the secretion of IL-6 and MMP3 by SF stimulated with ThCM (adalimumab, secukinumab) or BcCM (canakinumab). Moreover, cytokines released by lymphocytes activated signaling pathways that induce IL-6 and MMP3 secretion by SF which could not be suppressed by JAKi. Therefore, treatment with a combination of a JAKi and a bDMARD as therapy for individual patients could be an option.

## Data Availability

The data presented in this study are available on request from the corresponding author.

## Supplementary Materials

The following are available online at https://www.mdpi.com/article/10.3390/biomedicines9101413/s1, Figure S1: Viability of SF and Th cells treated with JAKi. Figure S2: Effects of tofacitinib, baricitinib, upadacitinib and bDMARDs on IFNγ (A), IL-17A (B) and IL-10 (C) expression by Th cells in mono-culture., Figure S3: Effects of tocilizumab, secukinumab, canakinumab or adalimumab on the expression of IDO1 by SF stimulated with ThCM.
