# Supplementary material for: Targeting of Janus Kinases Limits Pro-Inflammatory but Also Immunosuppressive Circuits in the Crosstalk between Synovial Fibroblasts and Lymphocytes
Source: Biomedicines. 2021 Oct 8;9(10):1413. doi: 10.3390/biomedicines9101413 (PMC8533088; doi:10.3390/biomedicines9101413)
Supplement: Supplementary file 1 [file biomedicines-09-01413-s001.zip › biomedicines-1381186-supplementary.pdf]

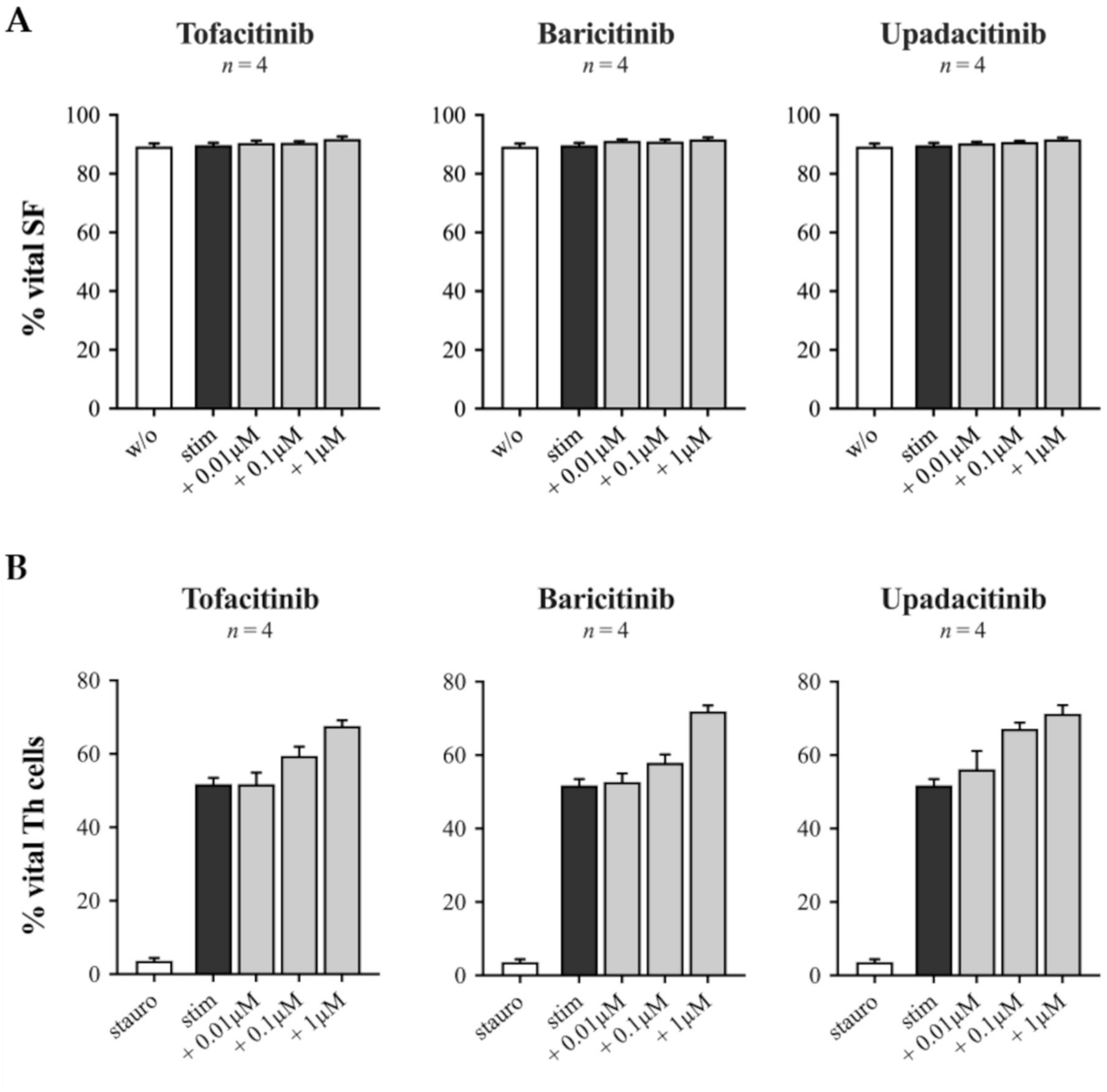

Figure S1: Viability of SF and Th cells treated with JAKi. (**A**) SF were stimulated with ThCM in the presence or absence of different concentrations of tofacitinib, baricitinib or upadacitinib. After 5 days of culture, cells were harvested, stained with Annexin V and propidium iodide and cell viability was determined by flow cytometry. (**B**) Th cells were stimulated with anti-CD3 and anti-CD28 antibodies and treated with different concentrations of JAKi as indicated. On day 6, Th cells were harvested and stained with Annexin V and propidium iodide. Treatment of Th cells with 2µM staurosporin (stauro) within the last 24 hours of culture served as a positive control for cell apoptosis. Data are shown as mean + SEM.

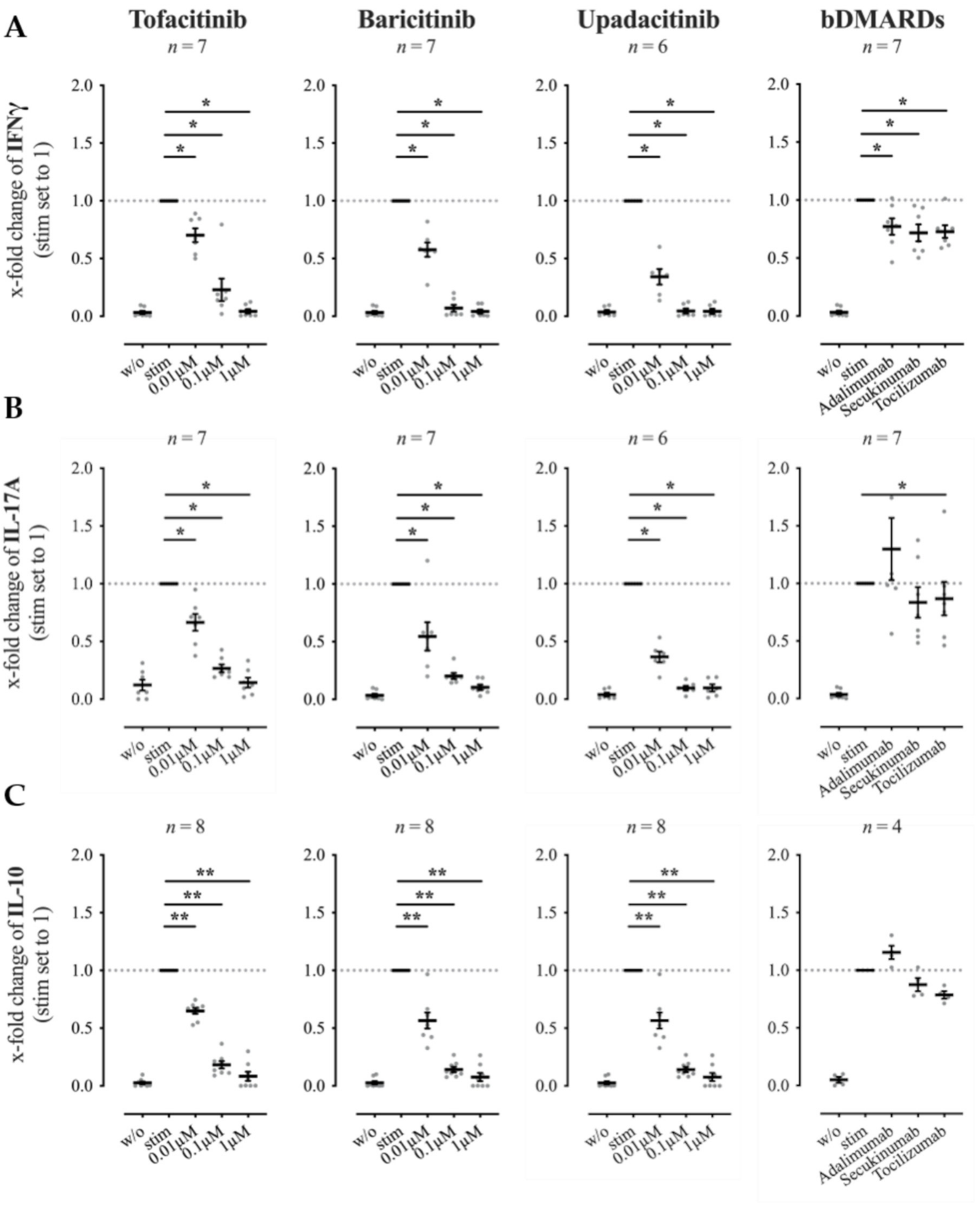

Figure S2. Effects of tofacitinib, baricitinib, upadacitinib and bDMARDs on IFNɣ (**A**), IL-17A (**B**) and IL-10 (**C**) expression by Th cells in mono-culture. Th cells were cultured with or without stimulation by anti-CD3/ anti-CD28 antibodies and treated with drugs as indicated. Supernatants were harvested on day 6 and cytokine concentrations were quantified by ELISA. Results are presented as x-fold change with stimulated Th cells set to 1 (mean concentrations ± SEM in pg/ml: IFNγ: 18068.31 ± 2731.63; IL-17A: 3033.54 ± 915.55; IL-10: 5194.79 ± 1416.66). BDMARDs shown were used at a concentration of 100 µg/mL. Data shown as mean ± SEM, significance tested using Wilcoxon signed-rank test, p** = <0.01, p* = <0.05.

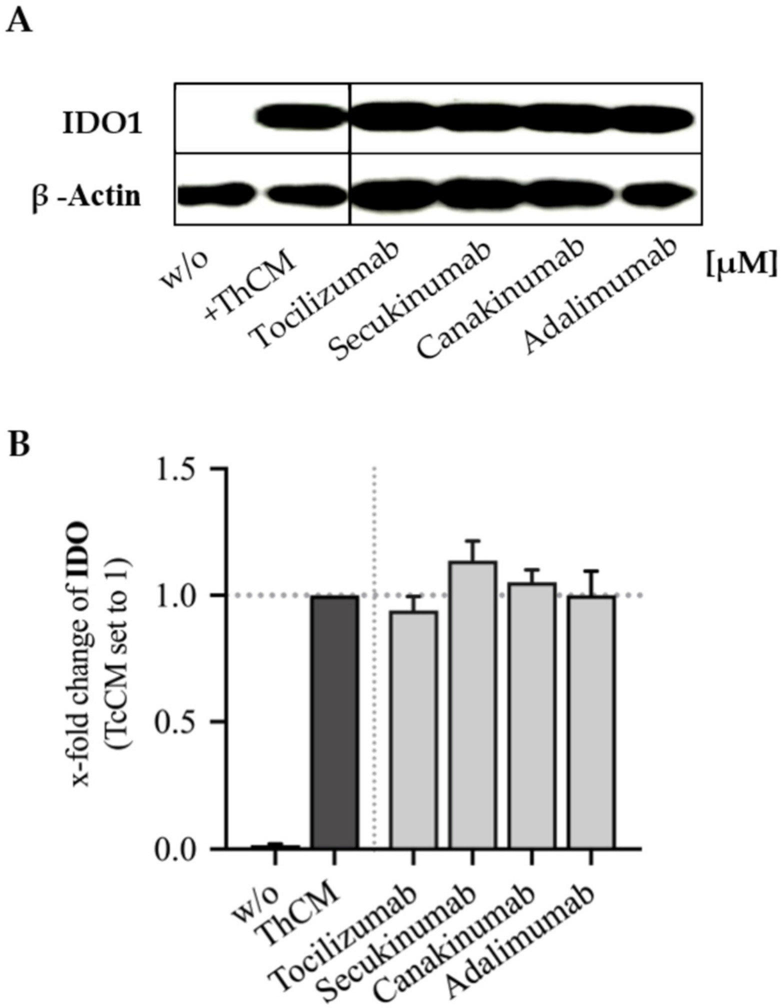

Figure S3. Effects of tocilizumab, secukinumab, canakinumab or adalimumab on the expression of IDO1 by SF stimulated with ThCM. SF were left untreated (w/o) or stimulated with ThCM and treated with tocilizumab ($n = 7$), secukinumab ($n = 12$), canakinumab ($n = 7$) or adalimumab ($n = 7$). On day 4, SF were harvested and whole cell extracts were subjected to western blot analysis. Shown are the results from one representative experiment (**A**) and the x-fold change of IDO1 relative to β-actin expression with SF stimulated with ThCM set as 1 detected in all experiments (**B**). Data shown as mean + SEM, significance tested using Wilcoxon signed-rank test.
